# Wild felids as sentinels of antimicrobial resistance: susceptible *Escherichia coli* isolates from a protected area in southeastern Brazil

**DOI:** 10.1007/s11259-026-11488-7

**Published:** 2026-08-28

**Authors:** Melrian Menezes Teixeira, Carolina Magri Ferraz, Hilton Entringer, Alessandra Figueiredo de Castro Nassar, Vanessa Castro, Marita Vedovelli Cardozo, Ana Carolina Srbek-Araujo, Gabriel Augusto Marques Rossi

**Affiliations:** 1 University Vila Velha (UVV), Av. Comissário José Dantas de Melo, n.21, Vila Velha, 29102-920 Espírito Santo Brazil; 2 Centro Para el Estudio de Sistemas Marinos, Centro Nacional Patagónico (CCT—CONICET), and Facultad de Ciencias Naturales y Ciencias de la Salud, Universidad Nacional de la Patagonia San Juan Bosco (UNPSJB), Puerto Madryn, U9120ACD Argentina; 3 Instituto Biológico (IB) de São Paulo, São Paulo, 04014-002 Brazil; 4 Department of Pathology, Reproduction and One Health, Faculty of Agricultural and Veterinary Sciences, São Paulo State University, (UNESP), Jaboticabal, São Paulo, 14884-900 Brazil

**Keywords:** AMR, Antibiotics, ESBL, Neotropical felids, Wildlife

## Abstract

Antimicrobial resistance (AMR) is a major global health concern, and its monitoring in wildlife is essential from a One Health perspective. Here, we obtained bacterial isolates and assessed the AMR profiles and extended-spectrum β-lactamase (ESBL) production of *Escherichia coli* isolated from fecal samples of wild felids in a protected Atlantic Forest area in southeastern Brazil. We collected 22 fecal samples and identified depositor species through trichological analysis (ocelot, *Leopardus pardalis* = 18; jaguar, *Panthera onca* = 1; puma, *Puma concolor* = 1; *Leopardus* sp. = 1; unidentified felid = 1). Bacterial isolation was performed using EMB agar and MacConkey agar supplemented with ceftriaxone or meropenem, and isolates were identified by MALDI-TOF MS. From these, 11 *E. coli* isolates (ocelot = 9; jaguar = 1; puma = 1) were selected to assess AMR using a standardized disk diffusion assay with 15 antimicrobials and to test for ESBL production. In addition to *E. coli*, the detected bacterial assemblage included *Achromobacter micicolens*,* Acinetobacter baumannii*, *Acinetobacter nosocomialis*, *Acinetobacter vivianii*, *Enterococcus faecalis*,* Ochrobactrum intermedium* and *Stenotrophomonas maltophilia*. All *E. coli* isolates were susceptible to all tested antimicrobials, and none showed evidence of ESBL production. These findings may suggest low selective pressure for AMR in the study area, reflecting limited circulation of antimicrobial-resistant bacteria. As free-ranging carnivores may act as sentinels of environmental change and integrate microbiota from prey, this study provides a baseline for monitoring AMR shifts across populations.

## Introduction

Antimicrobial resistance (AMR) is a major global public health threat that compromises treatment efficacy, prolongs hospital stays, and increases healthcare costs. The environmental dissemination of antimicrobial residues, antimicrobial-resistance genes (ARGs), and antimicrobial-resistant bacteria (ARBs) contribute to AMR spread across ecosystems. Wildlife is increasingly recognized as an important component of AMR epidemiology, reflecting environmental contamination and the interconnectedness of human, animal, and environmental health within a One Health framework (Di Francesco et al. 2025).

Among bacterial species, *Escherichia coli* is widely used in AMR surveillance due to its ubiquity in the intestinal microbiota of humans and animals and its ability to acquire and disseminate ARGs. Clinically important enzymes, including extended-spectrum β-lactamases (ESBLs) and carbapenemases, produced by some Enterobacterales such as *E. coli*, confer resistance to critically important β-lactams, including extended-spectrum cephalosporins and carbapenems. These organisms are often associated with multi-drug resistant (MDR) lineages due to the frequent co-occurrence of additional resistance determinants (Rossi et al. 2026) and are classified by the World Health Organization as a critical priority group.

Wild felids, occupying high trophic levels and thus feeding at higher positions within food webs, may be exposed to ARBs and ARGs acquired through contaminated prey, potentially reflecting AMR contamination across lower trophic levels and acting as sentinels of environmental AMR (Doyle et al. 2025). Non-invasive fecal sampling provides efficient access to bacterial communities associated with these predators and is particularly suitable for wildlife studies. This approach has been applied across carnivore taxa in both captive (Maly et al. 2025) and free-ranging settings (Gonçalves et al. 2013), providing reliable ecological insights even under remote field conditions. Nevertheless, studies on AMR in wild felids remain scarce, with research strongly biased toward the Northern Hemisphere (Vezeau and Kahn 2024; Smoglica et al. 2025). In this study, we characterized bacterial isolates from fecal samples of wild felids in a protected area in southeastern Brazil and assessed the antimicrobial resistance profiles and ESBL production of *E. coli*, thereby helping to fill key taxonomic and geographic gaps in wildlife AMR research.

## Materials and methods

**Fig. 1:**
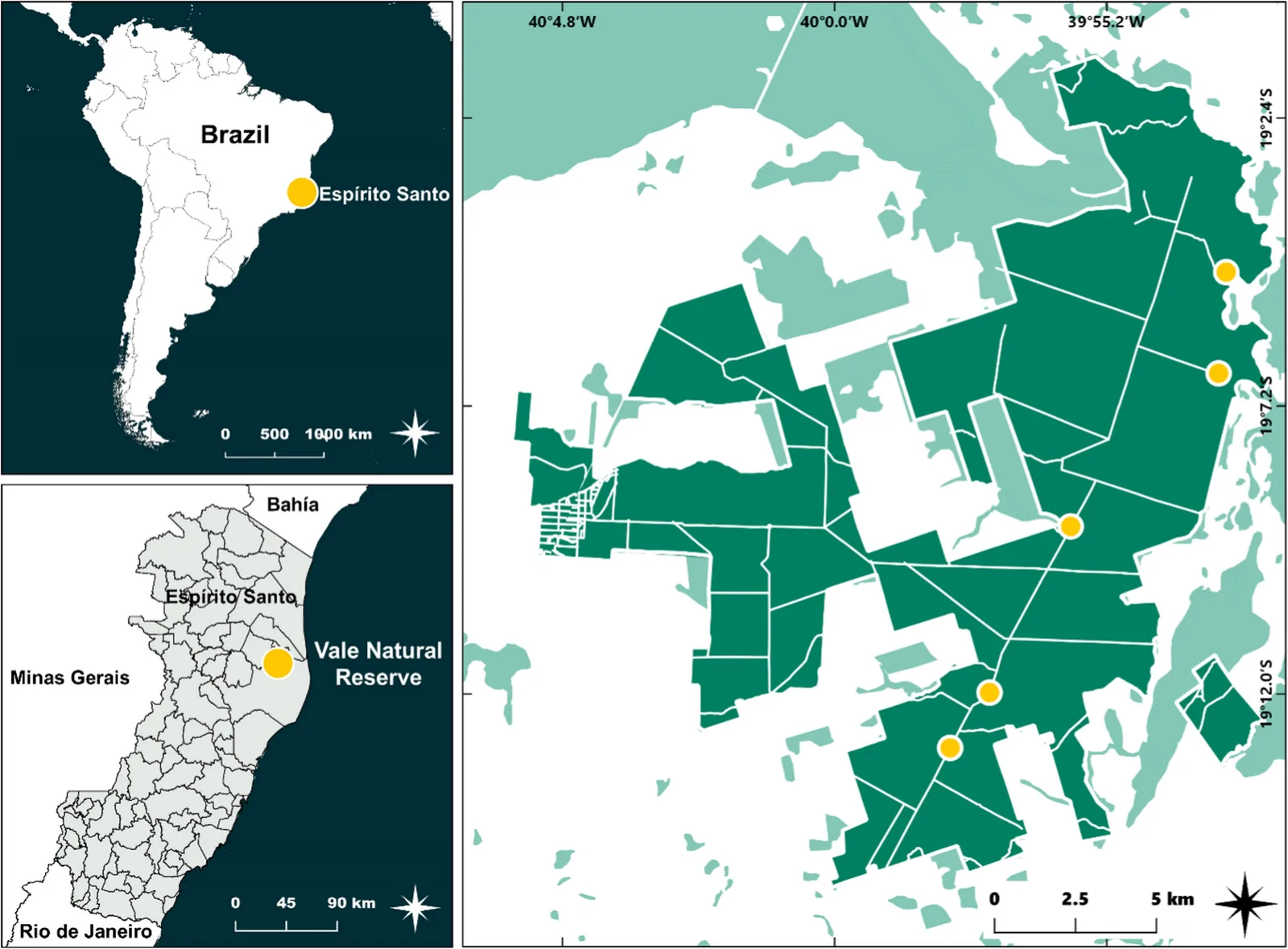
Location of the Vale Natural Reserve in Espírito Santo State, southeastern Brazil (left panels). The right panel shows the reserve area (dark green), including the internal road network (white lines) and sampling sites (yellow circles). Light green areas represent adjacent forest remnants, whereas white areas correspond primarily to anthropogenically altered landscapes, with minor representation of natural non-forest patches

The study was conducted in the Vale Natural Reserve (Reserva Natural Vale: RNV), located in the municipalities of Linhares and Sooretama, Espírito Santo, southeastern Brazil (Fig. 1). The reserve comprises approximately 22,711 hectares and, together with the Sooretama Biological Reserve (Reserva Biológica de Sooretama: RBS) and other adjacent private protected areas, forms a continuous forest block of nearly 53,000 hectares. This area represents approximately 11% of the remaining Atlantic Forest in Espírito Santo and maintains the original felid assemblage, including the jaguar (*Panthera onca*), puma (*Puma concolor*), ocelot (*Leopardus pardalis*), margay (*Leopardus wiedii*), southern tiger cat (*Leopardus guttulus*) and jaguarundi (*Herpailurus yagouaroundi*) (Srbek-Araujo et al. 2014). Despite its protected status, the RNV is embedded in a human-modified landscape dominated by agriculture and pastures (Kierulff et al. 2014). In addition, rivers entering the reserve traverse anthropogenically altered areas, and one of these watercourses receives effluent from the municipal wastewater treatment system of Sooretama, potentially facilitating external inputs.

Fecal samples were collected on multiple occasions between April 2023 and September 2024 through active searches conducted during walking surveys at five sampling sites within the RNV (four along internal roads and one in native shrub vegetation) (Fig. 1). The sites had been previously identified as suitable for fecal sample collection (Entringer Jr et al. 2022). Only fecal samples that were not visibly washed or degraded (still containing fecal material) were collected for microbiological analyses. Each sample was individually placed in a sterile plastic bag containing silica gel beads for desiccation, labeled with a unique code, and stored under cool, dry laboratory conditions until processing.

For bacteriological analysis, fecal samples were swabbed from the internal portion using sterile swabs to minimize external contamination, as environmental exposure can alter microbial composition and the internal core is less affected by external conditions (Maly et al. 2025). Subsequently, for felid species identification, the samples were disaggregated under running water using two nested fine-mesh sieves, and the retained material was then oven-dried. Predator guard hairs recovered from the retained material, which had been ingested during self-grooming, were identified by microstructural examination of cuticle and medulla patterns, allowing identification of the species responsible for fecal deposition (Srbek-Araujo et al. 2023).

In parallel, swab samples were inoculated onto three media: (1) Eosin Methylene Blue (EMB) agar for presumptive *E. coli* isolation; (2) MacConkey agar supplemented with ceftriaxone (4 mg/L) for screening extended-spectrum β-lactamase-producing enterobacteria; and (3) MacConkey agar supplemented with meropenem (4 mg/L) for screening carbapenemase-producing enterobacteria. Plates were incubated aerobically at 35 ± 2 °C for up to 48 h (Silva Rosa et al. 2026). All colonies with distinct morphologies were selected and identified by matrix-assisted laser desorption/ionization time-of-flight mass spectrometry (MALDI-TOF MS) using a Microflex LT/SH system (Bruker, USA). Isolates were preserved at − 20 °C in Brain Heart Infusion broth supplemented with 15% glycerol.

Antimicrobial susceptibility of *E. coli* isolates was evaluated by disk diffusion using doxycycline (30 µg), tetracycline (30 µg), gentamicin (10 µg), amikacin (30 µg), tobramycin (10 µg), imipenem (10 µg), meropenem (10 µg), ampicillin (10 µg), cefoxitin (30 µg), cefazolin (30 µg), cefuroxime (30 µg), fosfomycin (200 µg), ciprofloxacin (5 µg), sulfamethoxazole (27.75 µg) plus trimethoprim (1.25 µg), and chloramphenicol (30 µg) (Cecon, Brazil). Inhibition zone diameters were interpreted according to the breakpoints provided in the CLSI M100, Table 2A, which were used for AMR surveillance purposes (CLSI, 2024). *Escherichia coli* ATCC^®^ 25,922 was used as the quality control strain (CLSI 2024). ESBL production was investigated by the double-disk synergy test using ceftriaxone (30 µg), ceftazidime (30 µg), cefepime (30 µg), aztreonam (30 µg), and amoxicillin-clavulanic acid (30 µg).

## Results

A total of 22 wild felid fecal samples were analyzed, comprising ocelot (*n* = 18), jaguar (*n* = 1), puma (*n* = 1), *Leopardus* sp. (*n* = 1), and one unidentified felid. Of these, 15 were positive for bacteria. *Escherichia coli* was detected in 11 samples (ocelot = 9; jaguar = 1; puma = 1) and seven additional bacterial species were identified from eight samples (Table 1). *E. coli* isolates were susceptible to all antimicrobials tested, and none showed a phenotypic profile consistent with ESBL production.

**Table 1:** Bacterial isolates recovered from 22 fecal samples of wild felids collected in the Vale Natural Reserve, Espírito Santo, Brazil, between April 2023 and September 2024, across different culture media, including Eosin Methylene Blue agar (EMB), MacConkey agar supplemented with ceftriaxone (4 mg/L) (MAC + CRO), and MacConkey agar supplemented with meropenem (4 mg/L) (MAC + MPM)

Fecal sample	Felid	EMB	MAC + CRO	MAC + MPM
1	*Leopardus pardalis*	*Escherichia coli*	*-*	*-*
2	*Leopardus pardalis*	*Escherichia coli*	*Acinetobacter nosocomialis*; *Acinetobacter vivianii*	*Stenotrophomonas maltophilia*; *Enterococcus faecalis*
3	*Leopardus pardalis*	-	*-*	*-*
4	*Leopardus pardalis*	*Escherichia coli*	*-*	*-*
5	*Leopardus pardalis*	-	*-*	*-*
6	*Leopardus pardalis*	-	*-*	*-*
7	*Leopardus pardalis*	*Escherichia coli*	*-*	*-*
8	*Panthera onca*	*Escherichia coli*	*-*	*-*
9	Undentified felid	-	*Achromobacter micicolens*; *Stenotrophomonas maltophilia*	*Stenotrophomonas maltophilia*
10	*Leopardus pardalis*	-	*Acinetobacter baumannii*	*Stenotrophomonas maltophilia*; *Enterococcus faecalis*
11	*Leopardus* sp.	-	*Stenotrophomonas maltophilia*	*-*
12	*Leopardus pardalis*	-	*-*	*-*
13	*Leopardus pardalis*	-	*Ochrobactrum intermedium*	*-*
14	*Leopardus pardalis*	-	*-*	*-*
15	*Leopardus pardalis*	*Escherichia coli*	*-*	*-*
16	*Leopardus pardalis*	*Escherichia coli*	*Acinetobacter baumannii*	*Stenotrophomonas maltophilia*
17	*Leopardus pardalis*	*Escherichia coli*	*-*	*-*
18	*Leopardus pardalis*	*Escherichia coli*	*Acinetobacter vivianii*; *Enterococcus faecalis*; *Stenotrophomonas maltophilia*	*Stenotrophomonas maltophilia*
19	*Leopardus pardalis*	-	*-*	*-*
20	*Puma concolor*	*Escherichia coli*	*-*	*-*
21	*Leopardus pardalis*	*Escherichia coli*	*Stenotrophomonas maltophilia*	*Enterococcus faecalis*
22	*Leopardus pardalis*	-	*-*	*-*

## Discussion

To the best of our knowledge, only *E. coli*,* E. faecalis* and *S. maltophilia* have previously been reported in wild free-ranging felids. *E. faecalis* has been identified in fecal samples from free-ranging Geoffroy’s cats (*Leopardus geoffroyi*) in the Brazilian Pampa biome (Oliveira de Araujo et al. 2020) and Iberian lynx (*Lynx pardinus*) in Spain (Doñana National Park and Sierra Morena) (Gonçalves et al. 2013), whereas *S. maltophilia* has been associated with septicemia and death in a wild leopard (*Panthera pardus pardus*) in India (Agri et al. 2022). Although AMR was not assessed for these species, our findings expand current knowledge by documenting additional cultivable enteric microbiota members of wild felids, such as *A. micicolens*,* A. nosocomialis*,* A. vivianii*,* A. baumannii* and *Ochrobactrum intermedium*, and confirming taxa previously reported to exhibit AMR in other regions. However, we highlight that the time elapsed between fecal deposition and collection may influence the recovery of viable bacteria due to environmental exposure, thereby influencing the isolation of bacterial species. Consequently, the bacterial assembly described here should be regarded as a partial representation of the cultivable enteric microbiota present at the time of fecal deposition.

Similarly to our results, the absence of antimicrobial-resistant *E. coli* in fecal samples from the Amami rabbit (*Pentalagus furnessi*) has previously been documented in Japan (Matsunaga et al. 2020). The authors suggested that the exposure of this species to ARB originating from humans, livestock, or other wildlife populations has likely been limited (Matsunaga et al. 2020). This contrasts with reports from domestic and captive wild felids, in which antimicrobial-resistant *E. coli*, including ESBL-producing strains, has been documented (Furlan et al. 2020; Medina et al. 2024). These reports include ocelots and jaguars (Furlan et al. 2020; Medina et al. 2024). In Brazil, ESBL-producing *E. coli* has also been detected in free-living wildlife, including the creamy-bellied thrush (*Turdus amaurochalinus*) (Dalazen et al. 2023), black vulture (*Coragyps atratus*) (Carvalho et al. 2020), giant anteater (*Myrmecophaga tridactyla*) (Rossi et al. 2026), and coati (*Nasua nasua*) (Carvalho et al. 2020). In wildlife, AMR is generally linked to environmental exposure and anthropogenic impact (Rossi et al. 2026), suggesting that its occurrence is context-dependent and reflects local circulation at the human–animal–environment interface. Accordingly, although AMR can reach non-synanthropic species such as the Iberian lynx (*Lynx pardinus*) (Gonçalves et al. 2013), the absence of AMR in our study suggests a different scenario, likely influenced by local ecological conditions and exposure pathways.

Trophic interactions represent a plausible pathway by which wild predators may be exposed to ARBs and ARGs (Doyle et al. 2025). In the RNV, jaguars and ocelots exploit a wide range of prey (Entringer et al. Jr 2022; Santos et al. 2022). Exposure may be further increased by the consumption of domestic and synanthropic species, such as dogs (*Canis lupus familiaris*) (Entringer Jr et al. 2022), enhancing contact with anthropogenically influenced microbiota. Thus, these predators may serve as integrative sentinels because their trophic positions as either top predator (jaguar) or mesopredator (ocelot) enables them to integrate signals from multiple prey species and ecological processes through diverse exposure pathways.

The predominance of samples from ocelot over those from puma and jaguar is not unexpected, considering differences in population abundance and density, home-range size, habitat use, and detectability among these species. Indeed, ocelot is the most frequently recorded felid in the study area based on camera-trap surveys, including surveys conducted along the internal roads (Srbek-Araujo and Chiarello 2013) where part of the fecal samples was collected. Furthermore, as larger-bodied felids, puma and particularly jaguar generally occur at lower population densities and occupy larger home ranges than ocelot, which may reduce the probability of detecting and collecting fecal samples from these species. Unfortunately, no population estimates are available for pumas or ocelots in the study area. Population estimates are available only for jaguars within the Vale Natural Reserve (RNV), where the population has been estimated at 9 ± 1.98 SE individuals (95% CI: 9–17) (Srbek-Araujo and Chiarello 2017). Therefore, the observed distribution of fecal samples among species is likely explained by species-specific ecological differences rather than differences in sampling effort.

Despite its protected status and well-preserved area, the RNV is embedded in a landscape dominated by agriculture and cattle rearing (Kierulff et al. 2014) and connected to external areas through hydrological systems that may facilitate microbial influx. Although the water body receiving wastewater effluent from the municipality of Sooretama intercepts the southern portion of the reserve, the study area is crossed by an extensive drainage network, including both perennial and temporary watercourses that extend throughout the entire RNV (Kierulff et al. 2014). These watercourses also traverse anthropogenically altered landscapes surrounding the reserve, including rural settlements, villages, pasturelands, and agricultural areas. Therefore, there is no single potential point of entry for waterborne infectious agents into the forest fragment. These features may increase exposure of predators and prey to anthropogenic pressures, including ARBs. If the surrounding matrix strongly influences local food webs beyond prey composition, the absence of AMR among *E. coli* isolates may indicate that: (1) wild felids in the RNV experience limited exposure to ARBs and ARGs despite ecological conditions favoring trophic connectivity; or (2) ARBs are relatively uncommon in the surrounding landscape, at least at levels relevant for trophic transmission. This is noteworthy because *E. coli* is widely used as an indicator organism due to its ability to acquire and disseminate antimicrobial resistance determinants (Rossi et al. 2026). However, the absence of detected AMR should not be interpreted as its complete absence, as ARBs may occur at low frequencies or remain undetected due to the limited sample size. Within a One Health framework, the occasional presence of cattle (*Bos* sp.) and especially domestic dogs within the RNV (Srbek-Araujo et al. 2014) may facilitate transmission of ARBs between wild and domestic populations. These findings highlight the importance of maintaining the ecological integrity of the reserve and surrounding landscape to help limit AMR introduction and spread in wildlife and anthropogenic settings.

Our results help reduce taxonomic and geographic gaps in the characterization of bacteria associated with wild felids and provide baseline data for AMR surveillance in wildlife. Current knowledge remains biased toward a few model taxa and studies conducted in Europe (Vezeau and Kahn 2024; Smoglica et al. 2025), limiting comparative analyses across ecological contexts. Moreover, limited integration of ecological factors, such as diet, habitat use, and species interactions, restricts understanding of AMR circulation within wildlife communities (Vezeau and Kahn 2024; Smoglica et al. 2025). These limitations are further compounded by methodological inconsistencies and the lack of historical baselines, hindering the distinction between natural and anthropogenic AMR patterns (Vezeau and Kahn 2024).

In the RNV, the low resistance observed provides an important baseline for future studies and comparisons across ecological contexts. Strengthening this baseline will require longitudinal surveillance and integrative approaches, including bacterial taxa beyond *E. coli*, genomic tools, and parallel sampling of prey, environmental matrices, and domestic animals. Additionally, differentiating fecal samples according to individual animals through DNA analysis could help reduce potential biases. Such efforts are essential to clarify exposure pathways and the role of trophic interactions in AMR dynamics, reinforcing the importance of continuous wildlife surveillance within a One Health framework.

In conclusion, the absence of antimicrobial-resistant *E. coli* among wild free-ranging felids suggests low AMR-related anthropogenic pressure in the Atlantic Forest study area. Although this finding should be interpreted cautiously, considering the limited sample size, the possibility that multiple samples originated from the same individual, and the lack of information on the time elapsed between fecal deposition and collection. These results provide a valuable baseline for future comparisons across ecological contexts, contribute to the characterization of bacteria associated with wild felids, and highlight the value of protected environments and wildlife as sentinels of environmental AMR within a One Health framework. Continued surveillance integrating multiple bacterial taxa and environmental sources will be essential to better understand AMR circulation and transmission in natural ecosystems.

## Data Availability

The datasets generated during and/or analyzed during the current study are available from the corresponding authors on reasonable request.
